# Prevalence of Human Immunodeficiency Virus and Opportunistic Infections Among Transgender Patients in the Clinical Setting: An All-Payer Electronic Health Record Database Study

**DOI:** 10.1089/trgh.2019.0030

**Published:** 2020-09-02

**Authors:** Joshua D. Niforatos, Jonathon W. Wanta, Emily Durbak, Jacqueline Cavendish, Justin A. Yax

**Affiliations:** ^1^ Department of Emergency Medicine, The Johns Hopkins School of Medicine, Baltimore, Maryland, USA.; ^2^ Department of Psychiatry and Behavioral Sciences, University of Washington, Seattle, Washington, USA.; ^3^ Cleveland Clinic Lerner College of Medicine, Cleveland, Ohio, USA.; ^4^ Division of Population Health, Department of Emergency Medicine, University Hospitals Cleveland Medical Center, Cleveland, Ohio, USA.; ^5^ Department of Internal Medicine, University Hospitals Cleveland Medical Center, Cleveland, Ohio, USA.

**Keywords:** Explorys, HIV, opportunistic infection, transgender

## Abstract

We performed a cross-sectional analysis of the prevalence of HIV and opportunistic infections among transgender patients in clinical care. Of 10,160 transgender patients identified, 3.9% had a diagnosis of HIV, compared to 0.32% in the non-transgender cohort (*p*<0.0001). Transgender patients experience the burden of all opportunistic infection compared to non-transgender patients in this analysis, although prevalence of pneumocystis pneumonia was not significant. This cohort-based, all-payer electronic health record study of HIV patients connected to care revealed that transgender patients have a higher prevalence of HIV infection and opportunistic infections compared to the non-transgender cohort.

## Introduction

In the United States, ∼1.4 million adults identify as transgender.^1–3^ Methods to estimate the prevalence of human immunodeficiency virus in this population rely on questions from survey studies.^4–7^ The reported population prevalence of HIV in these studies ranges from 1.4% among all transgender persons to 3.4% to 21% among transgender women.^4,8^ However, these data may underestimate the actual prevalence of HIV in this population. For example, in one study, almost half of all the survey respondents did not know if they had ever been tested for HIV or the results of any previous HIV test.^4^ Thus, estimating the prevalence of HIV among transgender persons may benefit from alternative methods as opposed to survey study questionnaires and meta-analyses with significant heterogeneity.

We thus sought to estimate the prevalence of HIV among transgender persons in clinical settings using an all-payer electronic health record (EHR) database, which to our knowledge is the first study to use these methods.

## Materials and Methods

### Data source

We utilized the cloud-based Explorys, Inc. (Cleveland, OH) database. Extensive reporting on this database, including the full methodology and technical features, has been described elsewhere.^9,10^ The data in Explorys are collected from health records, laboratory systems, and billing inquiries.^10^ Currently, ∼60 million unique patient records from over 300 hospitals associated with 26 U.S. health care systems across all 50 states feed data from their respective EHRs to Explorys once every 24 h. Information from distinct EHRs is mapped into a single set of unified medical language system ontologies to standardize, normalize, and aggregate the data.^9^ More specifically, diagnoses and procedures for all patient records are mapped into Systematized Nomenclature of Medical Clinic Terms for clinical term (SNOMED-CT) hierarchies. SNOMED-CT collapses diagnostic codes from the international classification of diseases (ICD) into clinically meaningful, standardized categories using “umbrella” terms, while also documenting specific diagnoses, which allows researchers to utilize the web application's PopEx system to search for disease, procedures, and findings at the epidemiological level of a de-identified, aggregated patient cohort. SNOMED-CT diagnoses can be mapped back to ICD codes using the U.S. National Library of Medicine's Interactive Map-Assisted Generation of ICD Codes algorithm.

The Explorys database is compliant with both the Health Insurance Portability and Accountability Act (HIPPAA) and the Health Information Technology for Economic and Clinical Health (HITECH) Act. As a HIPPAA and HITECH compliant platform, information related to specific hospital systems in the Explorys database is not available to researchers. Finally, Explorys has been validated across numerous fields, including dermatology,^11^ endocrinology,^12,13^ neurology,^14^ gynecology,^15^ gastroenterology,^10^ orthopedics,^16^ surgery,^17^ and hematology.^9^ Use of Explorys has been deemed exempt from Institutional Review Board approval by University Hospitals Cleveland Medical Center.

### Cohort selection and definitions

We performed a retrospective analysis of the Explorys database for information collected between 1999 to April 2018. In this database, we selected all patients without missing demographic information. From this cohort, we identified an aggregated cohort of unique patients with a SNOMED-CT diagnosis of “transgender” at any time during the inclusion period, corresponding to previously published ICD-9 and ICD-10 classifications used to identify transgender patients in administrative claims databases (Supplementary Table S1).^18–21^ For the primary outcome of a diagnosis of HIV infection, we used the umbrella term “human immunodeficiency virus infection” to identify the prevalence of HIV, as previously described in a recent study.^22^ Other outcomes used diagnostic terms that correspond to their SNOMED-CT umbrella terms, such as “syphilis infection” and the specific opportunistic infections identified in Table 2. We compared these data to a non-transgender cohort that included all patients who had no transgender diagnosis during the study period and who had complete demographic information.

**Table 2.** Characteristics of Patients with History of HIV and Syphilis

Characteristics	Transgender cohort^a^	Non-transgender cohort^a^	p^*^
HIV demographics, *n* (%)	400 (3.9)^b^	170,870 (0.32)^b^	<0.0001
Sex			
Male	280 (70)	102,290 (60)	<0.0001
Female	120 (30)	68,380 (40)	
Race, *n* (%)			
African American	240 (60)	36,920 (22)	<0.0001
Caucasian	90 (23)	102,670 (60)	
Other	20 (5)	18,630 (11)	
Unknown	30 (8)	17,880 (10)	
Insurance status, *n* (%)			
Medicaid	210 (53)	56,410 (33)	<0.0001
Private	130 (33)	75,030 (44)	
Medicare	70 (18)	22,780 (13)	
Self-pay	40 (10)	17,960 (11)	
Syphilis demographics, *n* (%)	110 (1.1)^b^	35,340 (0.07)^b^	<0.0001
Male	80 (73)	19,530 (55)	0.0002
Female	30 (27)	15,810 (44)	

^*^ *p*-Values are from global χ^2^ tests.

^a^ Explorys reports data to the nearest 10. For this reason, percentages have been rounded and may not total 100.

^b^ Percent of total population is noted in the parentheses. Total population for transgender patients is 10,160. Total population for the non-transgender cohort is 53,449,400.

### Statistical analysis

Demographic data are presented as numbers and percentages. Chi-squared tests and Fisher's exact test were used to compare differences between groups. Given that Explorys rounds the number of patients to the nearest 10 for additional data protection, analyses were not conducted on outcomes that reported <10 patients as the data provided in Explorys is presented as “<10.” Statistical significance was set to *p*<0.05. All analyses were performed using IBM SPSS Statistics, version 25 (IBM).

## Results

We identified 53,449,400 patients without missing demographic data, of which 10,160 (0.019%) were transgender based on the aforementioned SNOMED search criteria. Table 1 lists baseline characteristics of transgender and non-transgender patients in the Explorys database. Transgender patients were primarily female (53.3%), Caucasian (65.5%), 35–39 years of age (interquartile range: 25–29, 54–59), and with private insurance (54.3%). Table 2 lists baseline characteristics of people living with HIV (PLWH) in the Explorys database. The prevalence of HIV infection was significantly higher in the transgender cohort (*n*=400) compared to the non-transgender cohort (*n*=170,870; 3.9% vs. 0.32%, *p*<0.0001). Demographic factors associated with HIV infection included male sex as classified in the EHR (*p*<0.0001), African American race (*p*<0.0001), and Medicaid insurance status (*p*<0.0001). Similarly, the prevalence of opportunistic infections was higher in transgender PLWH compared to the non-transgender cohort living with HIV, except for pneumocystis pneumonia (*p*=0.53; Table 3).

**Table 1.** Characteristics of Patients in the Explorys Database

Characteristics	Transgender cohort^a^	Non-transgender population^a^
	N=10,160	N=53,449,400
Sex		
Male	4740 (46.6)	24,259,090 (45)
Female	5420 (53.3)	28,987,100 (54)
Age, years (%)		
0–14	190 (1.9)	6,405,720 (12.0)
15–29	4490 (44.1)	9,192,020 (17.2)
30–39	2210 (21.7)	7,703,400 (14.4)
40–49	1200 (11.8)	7,149,080 (13.4)
50–59	970 (9.5)	7,506,600 (14.04)
60–64	490 (4.8)	3,621,180 (6.77)
>65	670 (6.6)	11,868,520 (22.2)
Median age, IQR	35–39 (25–29, 54–59)	40–44 (25–29, 60–64)
Race/ethnicity, *n* (%)		
Caucasian	6660 (65.5)	30,217,090 (56.53)
African American	1140 (11.2)	5,663,280 (10.60)
Hispanic/Latino	430 (4.2)	886,110 (1.66)
Asian	130 (1.2)	977,410 (1.83)
Other^b^	1800 (17.7)	11,991,870 (22.46)
Insurance type, *n* (%)		
Private	5520 (54.3)	20,879,650 (39.06)
Medicare	1030 (10.1)	5,965,830 (11.16)
Medicaid	2180 (21.4)	4,510,230 (8.44)
Self-pay	1380 (13.5)	7,180,500 (13.43)

^a^ Explorys reports data to the nearest 10. For this reason, percentages have been rounded and may not total 100.

^b^ Includes Asian, Pacific Islander, Native American, Alaskan Native, Latin American, Native Hawaiian, and unknown.

IQR, interquartile range.

**Table 3.** Opportunistic Infections and Immunization History Among Transgender and Non-Transgender Patients Living with HIV

	Transgender	Non-transgender	p^*^^,^^**^
	N=400	N=170,870	
Opportunistic infections, *n* (%)			<0.0001
Bacterial pneumonia	100 (25)	3740 (2.2)	<0.0001
Human papilloma virus	90 (23)	9610 (5.6)	<0.0001
Candidiasis			
Oral	60 (15)	8870 (5.2)	<0.0001
Esophageal	20 (5)	2440 (1.4)	<0.0001
Tuberculosis	30 (8)	19,670 (12)	0.012
Pneumocystis pneumonia	20 (5)	1190 (0.70)	0.53

^*^ *p*-Values are from global χ^2^ tests.

^**^ *p*-Values <0.05 were considered statistically significant.

## Discussion

To our knowledge, this is the first study to estimate the prevalence of HIV among transgender persons in a clinical setting using EHR data from an all-payer database. We identified 3.4% of transgender patients living with HIV, while previous survey studies have observed prevalence ranging from 1.4% to 14% in survey studies and meta-analyses of case series, respectively.^4,5,23^ We chose to estimate prevalence of HIV in transgender patients using EHR data in an attempt to mitigate some of the limitations associated with survey studies. The data captured in survey studies regarding HIV status most often provide the answer choices “Yes,” “No,” or “I don't know.”^4,5^ However, there are few reasons to question the prevalence of HIV from these studies, including (1) an estimated 15% of patients living with HIV in the United States do not know their serostatus^7,24^; (2) psychosocial factors and perceived importance of information affect memory of an individual's medical history^25^; (3) previous studies suggest inaccuracies are introduced when prevalence metrics rely on self-reported information^25–28^; and finally, (4) numerous studies have documented the phenomenon of nonacceptance of HIV as a coping mechanisms in vulnerable patients.^29–32^ For these reasons, there is sufficient susceptibility to underestimate disease prevalence in survey studies, particularly in a patient population with suspected increased baseline prevalence for HIV.

Our data confirm recent reports that transgender persons, including transgender women of color, have a higher prevalence of HIV compared to non-transgender persons.^3,5^ The highly cited 2015 Transgender Survey estimates the prevalence of HIV among transgender persons to be ∼1.4% among survey responds compared to the 3.9% documented in this study. However, over 40% of the participants in that survey did not know the results of a previous HIV test.^4^ Furthermore, while a greater proportion of transgender women in clinical care are living with HIV compared to the non-transgender cohort in this study (6.85% vs. 0.32%), a recent meta-analysis estimates the prevalence of HIV among transgender women to be around 21% in the United States. The aforementioned study, however, was limited by significant heterogeneity (*I*^2^=98.5%).^6^

While data are limited regarding the disease burden of opportunistic infections among this patient population, our study suggests that transgender patients living with HIV are more likely to have opportunistic infections compared to non-transgendered patients living with HIV. This increased burden of disease may reflect initial presentation as an AIDS-defining illness, poor adherence, or lack of access to antiretroviral therapy. Finally, we provide data that suggest an increased prevalence of syphilis infection in transgender persons, especially among those with a history of HIV, which has been noted in preliminary systematic reviews and randomized control trials of transgender persons on pre-exposure prophylaxis.^33,34^

Use of EHR data may limit the interpretation of prevalence of HIV among transgender patients throughout the United States for a few reasons. To begin, not all hospitals systems report HIV diagnoses or HIV testing to Explorys (personal communicating with IBM). In addition, we are limited by potential inconsistencies in documenting transgender identity in the EHR, which has been described extensively elsewhere.^2,35,36^ Given that the use of Explorys is both HIPPAA and HITECH compliant, we did not have access to individual patient charts to further explore either transgender or HIV documentation. Although it is possible that the Explorys database may not contain a representative sample of clinical settings where individuals seek care for HIV, the prevalence of PLWH in Explorys is 0.33%, which approximates the Centers for Disease Control and Prevention reported prevalence of 0.37% in the United States.^37^ Nevertheless, information from patients who receive screening through non-hospital systems, such as county health departments, stand-alone sexually transmitted disease clinics, and Free Clinics, are not included in the Explorys database.^37^ By addressing these limitations in the future, cloud-based EHR data could be used to frequently monitor the health needs of this minority patient population on a national level compared to primary reliance on survey studies that have significant limitations.

Despite the aforementioned limitations, this study of transgender patients in clinical care using a nationwide, all-payer EHR database provides evidence of a higher prevalence of HIV among transgender compared to non-transgender patients in the Explorys database.

## Supplementary Material

Supplemental data

## Abbreviations Used

EHR: electronic health record
HIPPAA: Health Insurance Portability and Accountability Act
HITECH: Health Information Technology for Economic and Clinical Health
IQR: interquartile range
PLWH: people living with HIV
SNOMED-CT: Systematized Nomenclature of Medical Clinic Terms for clinical term
