# Supplementary material for: Prevalence of Human Immunodeficiency Virus and Opportunistic Infections Among Transgender Patients in the Clinical Setting: An All-Payer Electronic Health Record Database Study
Source: Transgend Health. 2020 Sep 2;5(3):191–5. doi: 10.1089/trgh.2019.0030 (PMC7480718; doi:10.1089/trgh.2019.0030)
Supplement: Supplemental data [file Supp_Data.pdf]

Supplementary Data

Supplementary Table S1. ICD-9 Codes and Keywords Used to Identify Potentially Eligible Transgender Patients

ICD-9 diagnostic codes matching to current SNOMED ontology search terms	*ICD-10 diagnostic codes matching to current SNOMED ontology search terms*	*SNOMED ontology search terms*
302.5-Transsexualism	F64 Gender identity disorders	Transgender
302.50-Transsexualism with unspecified sexual history (a.k.a. "transsexualism not otherwise specified")	F64.0 Transsexualism	Transsexual
302. 51-Transsexualism with asexual history	F64.1 Dual role transvestism	Transvestite
302.52-Transsexualism with homosexual history	F64.2 Gender identity disorder of childhood	Gender identity disorder of adulthood
302.53-Transsexualism with heterosexual history	F64.8 Other gender identity disorders	Gender identity disorder of adolescence
302.85-Gender identity disorder in adolescents or adults	F64.9 Gender identity disorder, unspecified	Gender identity disorder of childhood
302.6-Gender identity disorder in children		Gender dysphoria
302.3-Transvestic fetishism		

ICD-9 codes used in this study come from previous identified and validated codes from studies of administrative claims databases.[S1–S4]
ICD, international classification of diseases; SNOMED, Systematized Nomenclature of Medical Clinic Terms.

Supplementary References

S1. Downing J, Conron K, Herman JL, Blosnich JR. Transgender and cisgender US veterans have few health differences. Health Aff (Millwood). 2018;37: 1160–1168.

S2. Dragon CN, Guerino P, Ewald E, Laffan AM. Transgender Medicare beneficiaries and chronic conditions: exploring fee-for-service claims data. LGBT Health. 2017;4:404–411.

S3. Proctor K, Haffer SC, Ewald E, et al. Identifying the transgender population in the Medicare program. Transgender Health. 2016;1:250–265.

S4. Progovac AM, Cook BL, Mullin BO, et al. Identifying gender minority patients' health and health care needs in administrative claims data. Health Aff (Millwood). 2018;37:413–420.
